# Supplementary material for: Vertical Mental Timeline Is Not Influenced by VisuoSpatial Processing
Source: Brain Sci. 2024 Feb 17;14(2):184. doi: 10.3390/brainsci14020184 (PMC10886795; doi:10.3390/brainsci14020184)
Supplement: Supplementary file 1 [file brainsci-14-00184-s001.zip › brainsci-2834624-supplementary.pdf]

Article

Vertical Mental Timeline Is Not Influenced by VisuoSpatial Processing

Alessia Beracci [1] and Marco Fabbri [2,*]

[1] Department of Psychology Renzo Canestrari, University of Bologna, 40126 Bologna, Italy; alessia.beracci@unibo.it
[2] Department of Psychology, University of Campania Luigi Vanvitelli, 81100 Caserta, Italy
* Correspondence: marco.fabbri@unicampania.it; Tel.: +39-0823275333

Supplementary Materials

The Figure S1 displays the mean RT difference (dRT) between up and down keys (RT up key – RT down key) for each temporal word defined by the specific order sequence reported in the corresponding Time-to-Position task in each experiment. The word order in each Time-to-Position task for every experiment is displayed in the Appendix A.

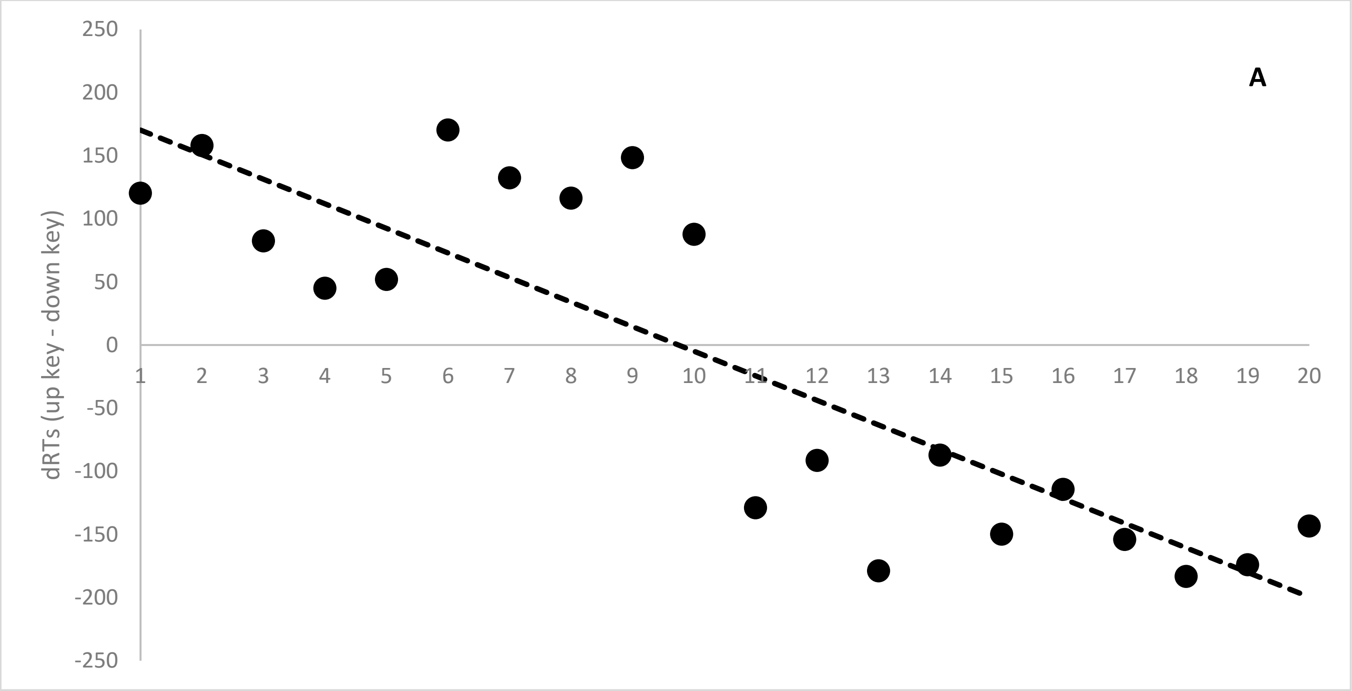

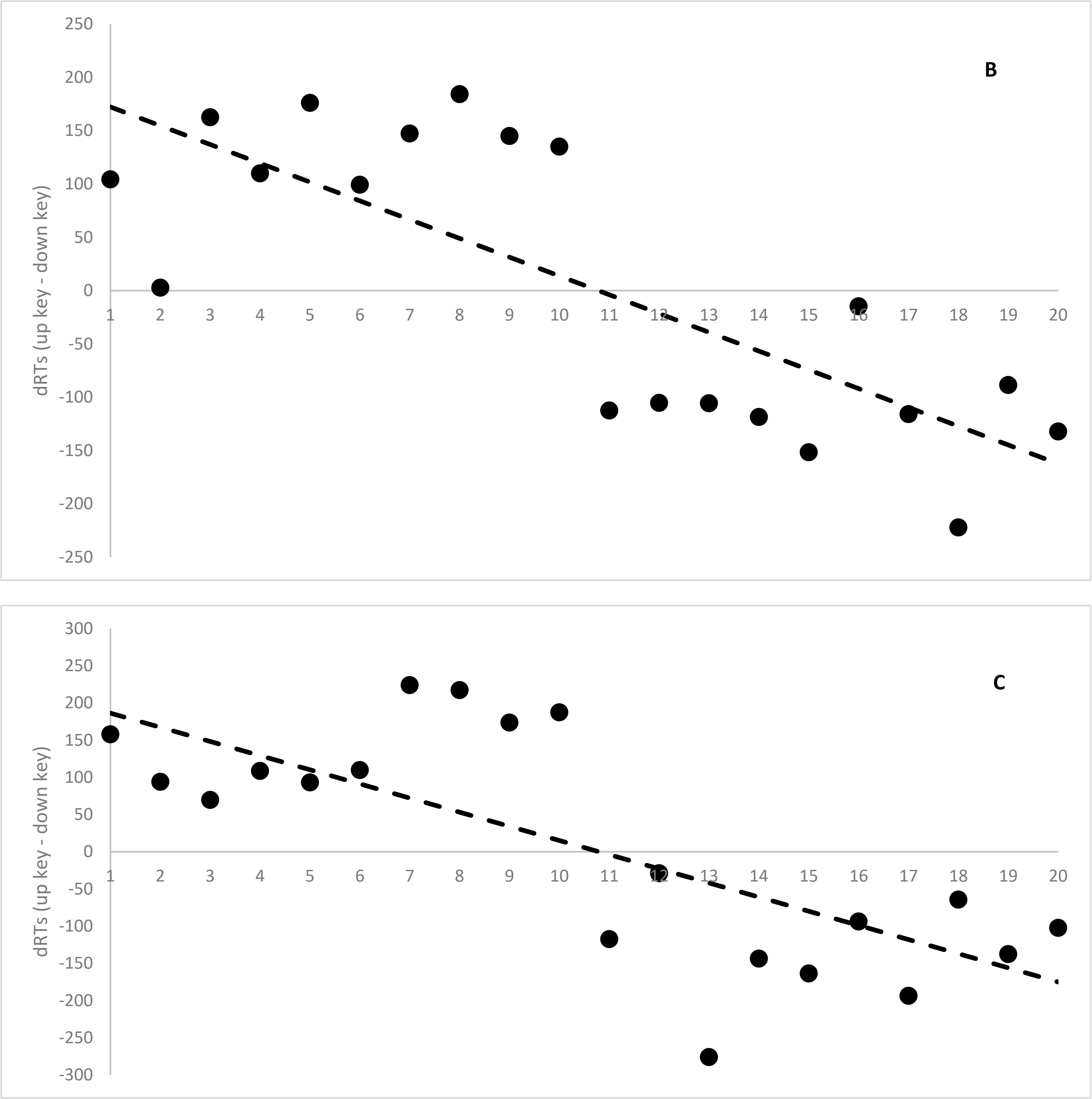

Figure S1. The dRTs between up and down keys for each temporal word (1-20) of the Experiment 1 (a), Experiment 2a (b) and Experiment 2b (c). The word order in each figure is displayed in the Appendix A.

A deep inspection of the Figure S1 reveals that a binary (or categorical) model could also explain the trend displayed. To test which was the better description of the regression model, we calculated the root-mean-squared-error (RMSE) for a linear (or continuous) and binary split (categorical) model in each experiment. Specifically, we calculated the square-root of the sum of the squared deviations between all the observed data and the predicted data according to the regression equation for the linear model (RMSE_lin). On the opposite, the square-root of the sum of the squared deviation between all the data for past words and their mean value plus all the squared deviations between the data for future words and their mean value was calculated for the binary split model (RMSE_bin). The Table S1 reports these RMSE values for each model in every experiment. In each experiment, the RMSE_bin value was lower than that of the RMSE_lin, and generally the RMSE values were lower in the Experiment 1 respect to other experiments.

	RMSE_lin	RMSE_bin
Experiment 1	67.75	52.61
Experiment 2a	83.26	69.83
Experiment 2b	102.61	84.35

An additional analysis was performed to assess the relationship between speeded binary and Time-to-Position tasks, as well as to test the continuous or categorical form of the STEARC effect [27]. Specifically, we tested whether the RT differences were predicted by time (past vs. future), Distance in the Time-to-Position task, and their interaction (see Beracci et al. [27] for a description of the analysis). For the Experiment 1, the maximum model with the categorical predictor (TimeC), the continuous predictor Distance, and their interaction with random slopes and intercepts on the dRTs showed a significant TimeC effect (p = .0001), while no Distance effect was observed (p = .22). Their interaction was significant (p = .01). Analyzing the minimum model with both main effects only confirmed the TimeC effect (p = .0001) and the lack of a significant effect of Distance factor (p = .23). The best fitting model (Table S2) was: modelmax < -LMER(UminusD ~ TimeC + Distance +TimeC*Distance + (1 + TimeC|SubjectID). As regards the Experiment 2a, the maximum model failed to converge, while the maximum model without random slope on dRTs showed a significant TimeC effect (p = .008), no Distance effect (p = .36), and a significant interaction between predictors (p = .00001). The minimum model with both main effects confirmed a significant TimeC effect (p = .001), but not a significant Distance effect (p = .41). The best fitting model (Table S2) was: modelmax < - lmer(UminusD ~ TimeC + Distance + TimeC*Distance + (0 + TimeC | SubjectID). Finally, for the Experiment 2b, we basically replicated the previous results of the Experiment 2a with a failure of model convergence with random slopes and intercepts in the maximum model, while the TimeC predictor was significant (p = .01) and the interaction between predictors (p = .05) tended towards the significance (and the Distance effect was not significant with p = .43), when the model was without random slope. The minimum model with both main effects showed a tendency towards the significance for the TimeC (p = .05) and no Distance effect (p = .43). The best fitting model was: modelmin < -lmer(UminusD ~ TimeC + Distance + (0 + TimeC | SubjectID). Altogether these results suggested that the STEARC effect displayed in the regression was categorical but the interaction between categorical and continuous predictors could indicate that the mental representation of time was continuous.

Table S1. Comparisons of the two models for each experiment. In the Table the Aikake Information Criterion (AIC), Bayesian Information Criterion (BIC), LogLikelihood (LL), Df (degrees of freedom) are reported in addition to the number of parameters, the chi-squared (χ^2) value and its p value.

	**Models**	**Number of parameters**	**AIC**	**BIC**	**LL**	**Deviance**	χ^2	**Df**	p
**Experiment 1**	Minimum	16	16066	16147	-8017.2	16034			
	Maximum	22	16062	16172	-8008.9	16018	16.729	6	.01
**Experiment 2a**	Minimum	14	12412	12478	-6191.9	12384			
	Maximum	20	12372	12467	-6166.1	12332	51.638	6	.0001
**Experiment 2b**	Minimum	14	13102	13169	-6537.0	13074			
	Maximum	21	13103	13203	-6530.3	13061	13.403	7	.063
